# A randomized controlled trial of mindfulness: effects on university students’ mental health

**DOI:** 10.1186/s13033-023-00604-8

**Published:** 2023-10-13

**Authors:** Giovana Gonçalves Gallo, Daniela Fernandez Curado, Mayra Pires Alves Machado, Marília Ignácio Espíndola, Vitor Villar Scattone, Ana Regina Noto

**Affiliations:** Departamento de Psicobiologia, NEPSIS – Núcleo de Pesquisa em Saúde de Uso de Substâncias – MBRP Brasil – Centro Brasileiro de Pesquisa e Formação em Prevenção de Recaídas Baseada em Mindfulness (MBRP), Universidade Federal de São Paulo, São Paulo, 04023-062 SP Brazil

**Keywords:** Anxiety, Depression, Stress, Insomnia, Intervention

## Abstract

**Background:**

The development of mental health disorders is common in the university population, and mindfulness-based interventions (MBIs) seem to be effective in addressing them in different contexts. Thus, this study investigated the impact of an 8-week MBI adapted to university students from the Mindfulness-Based Relapse Prevention (MBSR) on different symptoms related to mental health problems, specifically symptoms of anxiety, depression, stress and insomnia.

**Methods:**

University students (n = 136) were randomized into MBI group (n = 71) or wait-list group (n = 65). All participants completed self-administered questionnaires before and after the intervention, and the experimental group answered questionnaires weekly during intervention. Generalized mixed models were used to assess the effects of the intervention.

**Results:**

There were improvements in the symptoms of stress (B = 5.76, p < 0.001), depression (B = 1.55, p < 0.01) and insomnia (B = 1.35, p = 0.020) from the beginning of the intervention to the final assessment when it was compared to the control group. No effect was found in respect of trait anxiety. The MBI was found to be effective in reducing important symptoms related to university students’ mental health, possibly grounding further research on the intervention’s potential of preventing the development of mental disorders.

**Trial registration:**

The research was registered in the Brazilian Registry of Clinical Trials (ReBEC) - number RBR-63qsqx, approved at 09/16/2019.

## Background

The mental health of university students has been a topic of growing interest for researchers and psychologists worldwide. High rates of mental disorders have been reported in this population [1, 2], leading to an increase in the demand for mental health services at universities [2–4] and a search for effective ways to meet this demand. When it comes to symptoms of anxiety and depression, the American College Health Association (ACHA) reported that 41.9% of students had felt so depressed at some time in the previous year that it was difficult to function, and 63.4% reported overwhelming anxiety during the same period [5].

In Brazil, according to a study by the National Association of Directors of Federal Institutions of Higher Education (Andifes) in 2018, about 30% of undergraduate students in federal institutions had sought psychological care in the previous two years and more than 10% took psychiatric medication [6]. In addition to being among the health problems that most impact the lives of individuals [7], anxiety and depression disorders also share symptoms, such as fatigue, problems related to sleep and irritability, and cognitive processes, such as negative affects and errors in processing information [8].

Impaired sleep is another aspect of this group’s mental health that deserves attention [9, 10], especially when considering it has been previously shown to be associated with physical and other mental health problems [11]. It has also been suggested that the high levels of stress experienced by some students may have a negative impact on sleep, changing its length and its quality [10, 12–14]. Moreover, high levels of stress can lead to several other negative consequences related to hormones, and it has been increasingly experienced by students over recent decades [15, 16].

Thus, it becomes clear the association among these symptoms, as well as the high need to find methods to promote students’ well-being and improve their mental health. In this regard, mindfulness-based interventions may be an important tool to be better explored, based on recent years reports of positive results in different settings. Mindfulness can be defined as the ability to “pay attention at the present moment in an intentional and non-judgmental way” [17] and has become popular in recent decades, presenting very positive results when associated with psychotherapy for the treatment of depression and anxiety disorders [18–20].

Mindfulness-based interventions (MBIs) have been proven to be effective in a wide range of problems [21], such as in stress reduction (using mindfulness-based stress reduction - MBSR) [22–24], in treating sleep-related problems [25–27], anxiety and depression disorders [28, 29] (using the mindfulness-based cognitive therapy protocol - MBCT) and also as a therapeutic adjunct in treating people with alcohol use disorders [30] (with the use of mindfulness-based relapse prevention protocol - MBRP). The latter stands out as a protocol that develops awareness of triggers and of internal reactions to stimuli, promoting more skilled responses and favouring the reduction of reactive behaviours, such as the use of substances in people with substance use disorders [31].

When it comes to its effects in university students, literature already provides some very important benefits brought by MBIs, especially when targeting those with an already diagnosed mental disorder [32]. Similarly to what was observed in other contexts, MBIs seem to promote an improvement of distress, anxiety and depression symptoms, well-being and rumination on university students [33].

However, more than being used as an adjunct treatment, the techniques could serve a greater purpose if also offered as a possibility of psychoeducation and self-care for those with or without mental disorders’ symptoms, aiming at preventing the emergence or aggravation of symptoms and future development of mental disorders. This approach of MBIs is not commonly reported in literature but might be a promising aspect to be evaluated. Therefore, the present study aimed at analysing the impacts of MBI on symptoms of anxiety, depression, stress and insomnia in university students and explore the path of stress, depression and insomnia throughout the intervention. Our initial hypothesis was that MBIs could improve mental health, represented by a decrease in the scores of instruments used to measure the cited symptoms.

## Methods

### Study design

This is a randomized controlled clinical trial undertaken in person in two Brazilian universities. It is part of a larger study entitled ‘Risk prevention based on mindfulness for young populations in educational contexts’ (CNPQ (nº426092/2018-0) and was registered in the Brazilian Registry of Clinical Trials (ReBEC), registration number RBR-63qsqx – submission date: 20/02/2019.

### Participants

The inclusion criteria were: Undergraduate or graduate students aged over 18 enrolled at any Brazilian university, interested in the objectives of the study and who agreed to participate in the research. The exclusion criteria were: the presence of self-reported psychotic disorder, severe cognitive impairment or having already undertaken mindfulness therapy.

A total of 136 participants were recruited and randomized, of whom 76 finished the study (55.8%) taking part on the second application of questionnaires (T1). Of those, 37 were from the experimental group and 39 from the control group. Students from any university were accepted as long as they could participate in the mindfulness sessions at one of the two public universities where the study was developed. There were no exclusions of volunteers from the research and reasons for dropout were not collected. Dropouts from the experimental group did not complete more than 4 sessions out of 8 or did not answer the questionnaires by the end of the research. Dropouts from control group did not take part in the second application of questionnaires but were also offered the opportunity to participate in the MBI offered after.

### Procedures

**Fig. 1:**
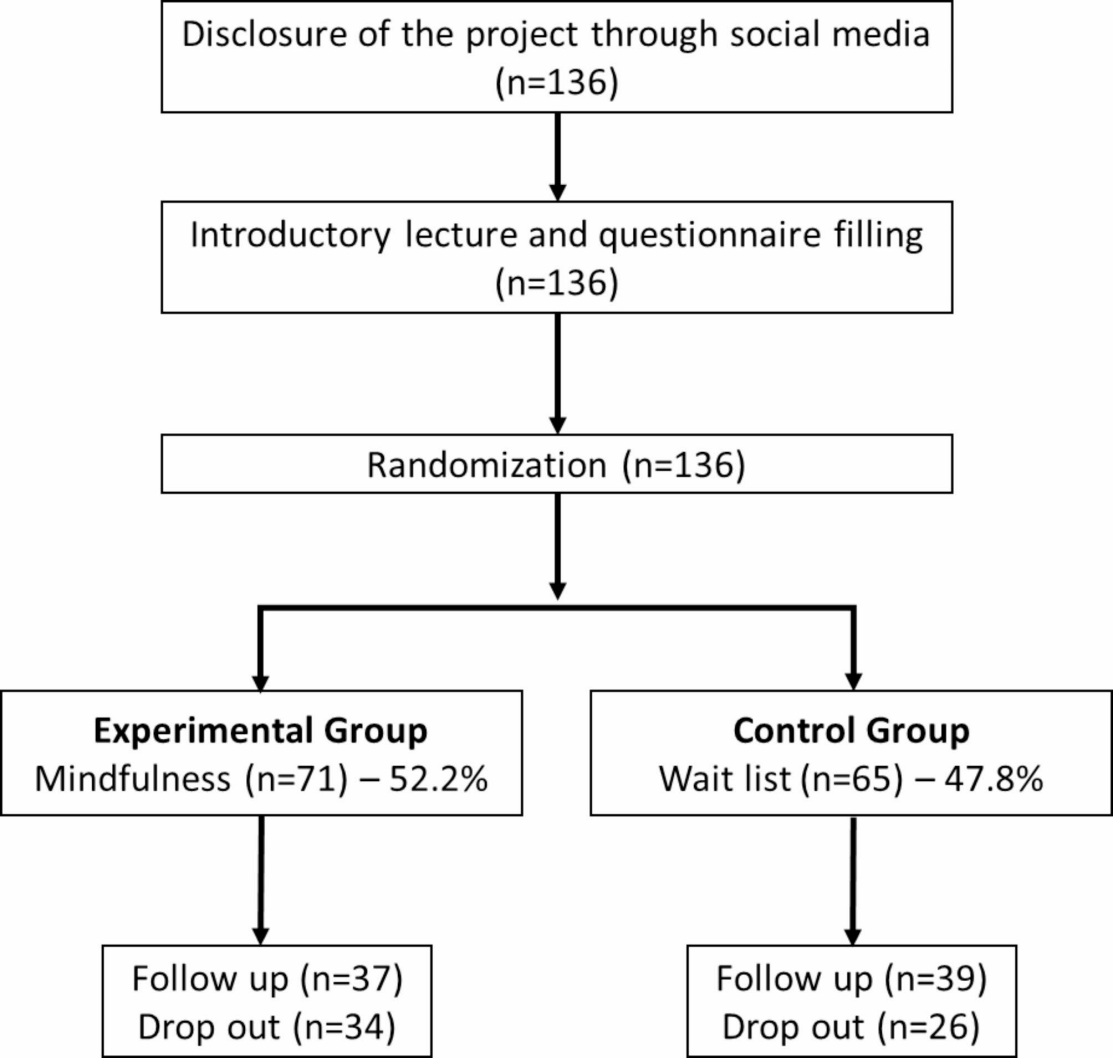
Flowchart of research participants

Volunteers were recruited through social media and an initial presentation was made in person to clarify the research procedures and objectives. After signing an Informed Consent Form in person, data collection began with the participants completing the instruments individually, supervised by a member of the research team. The data collected at this point was considered baseline (T0). The participants were then randomly assigned to either the experimental group or a wait-list control group through computer-based generation of numbers in blocks, considering the 12 different groups that were conducted throughout the year. In the following week, the experimental group started the MBI 8-weeks protocol, during which they filled in the instruments PHQ-9, ISI and PSS-10 weekly, at the beginning of each session. After eight weeks, all volunteers were asked to complete all questionnaires again (T1). The STAI questionnaire was not applied weekly due to its stability characteristic, therefore anxiety was only measured at the beginning and by the end of the intervention. The flowchart of the study procedures is described in Fig. 1. The project was approved by the Federal University of São Paulo Research Ethics Committee, number 3.201.133 (CEP/UNIFESP n: 0127/2019).

### Measures

#### Sociodemographic data questionnaire

a questionnaire inquiring age, sex, psychological disorders, education, and parents’ education. This questionnaire was answered only once, at the beginning of the participation, by each volunteer.

#### PHQ-9 (Patient Health Questionnaire – 9)

A 9-item questionnaire evaluating the frequency of the participants’ depressive symptoms in the past week, scored using a Likert scale ranging from 0 (none) to 3 (almost every day) validated in Brazil [34, 35]. Participants who were in the intervention group answered it weekly, as well as at T0 and T1 alike the control group.

#### Perceived stress scale (PSS-10)

A 10-item scale with responses based on a Likert Scale ranging from 0 (never) to 4 (always) that evaluates the frequency of stressful situations in the past month. The Brazilian validated version (36) was used. Participants who were in the intervention group answered it weekly, as well as at T0 and T1 alike the control group.

#### Insomnia Severity Index (ISI)

A 7-item questionnaire evaluating insomnia symptoms and its influence on daily life, measured by responses on a Likert scale ranging from 0 to 4, validated in Brazil [37]. Participants who were in the intervention group answered it weekly, as well as at T0 and T1 alike the control group.

#### State-trait anxiety index (STAI)

A 20-item instrument that evaluates anxiety symptoms using a 4-point Likert scale, from 0 - “almost never,” to 4 - “almost always”. In this study, only the trait subscale was used, since it is less sensitive to environmental change. Higher scores represent more symptoms, and the scale used had already been validated in Brazil [38]. The questionnaire was answered only at T0 and T1 by both groups.

### Mindfulness based intervention (MBI) protocol

An adaptation of the MBRP (Mindfulness Based Relapse Prevention) protocol was used, following the parameters given by Crane (2016) [39]. In this adaptation, the focus shifted from preventing relapses to managing reactivity, and practices’ time and session itself were reduced from 2 h to an hour and a half. The MBRP was chosen to be the protocol adapted from due to the identified necessity and possibility of better addressing reactivity, which impacts students’ mental health, while providing them with techniques of health promotion and gathering the benefits that have been observed as a consequence of taking part in MBI protocols.

The intervention happened weekly for 8 weeks in groups composed by 3 to 10 participants each, all facilitators had a professional qualification in mindfulness and had previous experience conducting the protocol. Following the same pattern as the original protocol, sessions had as main theme, in order: (session 1) mindfulness in daily life and autopilot; (session 2) awareness of challenges, sensations, thoughts and reactions; (session 3) mindfulness in daily life; (session 4) mindfulness in challenging situations; (session 5) acceptance and skilful action; (session 6) seeing thoughts as thoughts; (session 7) self-care and balanced lifestyle; and (session 8) social support and continuation of practice. Also, participants were encouraged to practice mindfulness at home, with both the formal and informal techniques discussed throughout the sessions.

### Data analyses

The analyses were performed using RStudio software version 1.2.5019, considering a significance level of 5%. Packages used were glmmTMB [40] and siPlot [41]. For sample characterization, categorical variables were presented as absolute frequency and percentage. For analyses of weekly changes, all models considered linear distribution and time as a fixed effect. To consider the dependence effect between the temporal measures, the subjects’ ID was included as a random effect. Interaction effects were represented in line graphs.

Generalized mixed models were used to assess the effects of MBI on the outcomes (anxiety, depression, stress and insomnia). As the outcomes were measured from the sum of items evaluated on Likert scales, three possible discrete distributions were tested for the dependent variables: normal, Poisson and negative binomial. The choice of the best distribution was based on adherence criteria, using the lower Akaike Information Criteria (AIC) [42].

All models considered as fixed effects group, time and the interaction between both. The control variables were sex, age and whether or not students were in exam periods. To consider the effect of dependence between temporal measures, the subjects’ id was included as a random effect, allowing variation of the intercept between individuals. The analyses were conducted among the subjects who did not have missing values in the variables used in each model (there was a 0,76% missing out of the total amount of items of questionnaires). The interaction effects were represented in line graphs, containing the average of the outcome weighted by covariate levels.

## Results

Among those who completed the questionnaires at T1, 61 were women (80.3%) and 15 men (19.7%), with an average age of 25 years (± 6.2 SD), ranging from 18 to 41 years. Most of the participants were undertaking undergraduate courses (61.3%), rather than postgraduate courses (38.7%), and 84.2% of participants studied at public universities. The description of all randomized participants can be found in Table 1.

**Table 1:** Sample description

			Experimental Group		Control Group		
	n	%	n	%	n	%	
Sex (n = 136)							
Female	111	81.6	54	76.1	57	87.7	
Male	25	18.4	17	23.9	8	12.3	
Age group (n = 135)							
18 to 24 years old	75	55.6	43	61.4	32	49.2	
25 to 30 years old	32	23.7	14	20.0	18	27.7	
Over 30 years old	28	20.7	13	19.6	15	23.6	
Education (n = 135)							
Undergraduate student	83	61.5	45	64.3	38	58.5	
Graduate student	52	38.5	24	35.7	28	41.5	
University (n = 136)							
Public	117	86.0	57	80.3	60	92.3	
Private	19	14.0	14	19.7	5	7.7	
Self-reported disorder							
Anxiety	13	9.6	7	9.9	6	9.2	
Depression	3	2.2	2	2.8	1	1.5	
Anxiety and Depression	11	8.1	8	11.3	3	4.6	
Does not present	29	21.3	16	22.5	13	20.0	
Did not declare	80	58.8	38	53.5	42	64.6	

**Fig. 2:**
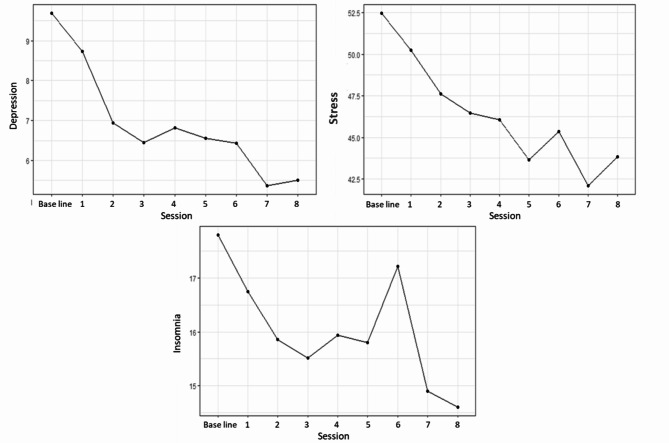
Progression of the average scores of PHQ-9 for depression, PSS-10 for stress, and ISI for insomnia, for students undertaking weekly mindfulness sessions

**Fig. 3:**
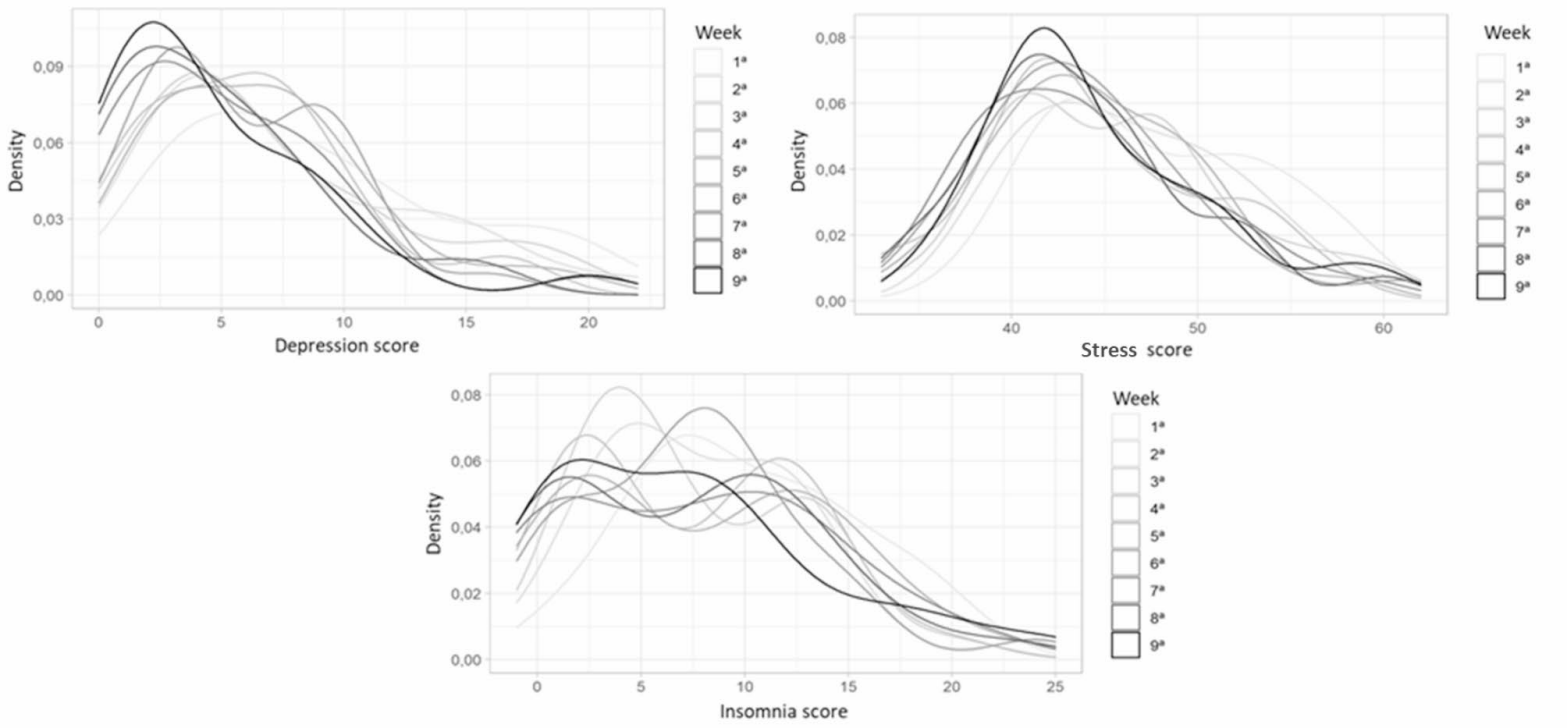
Histograms of weekly depression (measured by PHQ-9), stress (measured by PSS-10) and insomnia (measured by ISI) scores distribution of intervention group

The symptoms analysed weekly throughout the intervention (stress, depression and insomnia), when compared to baseline, showed a significant decrease in all sessions (Table 2). The progression of the average scores (Fig. 2) makes it clear that over the first three sessions there was a continuous decrease in the averages, with a slight increase in symptoms of stress and depression after the fourth session. In the fifth session all symptoms decreased again, but after the sixth session there was a peak of symptoms of stress and insomnia (still below the initial mean). Finally, the mean scores decrease again, with symptoms of stress showing its lowest value in the seventh session and symptoms of insomnia in the eighth. At Fig. 3 it is possible to see the score distribution of all three symptoms over the protocol.

**Table 2:** Weekly changes in symptoms of intervention group

Insomnia	B	[95% IC]	p
Time (reference = baseline)			
Session 1 Session 2 Session 3 Session 4 Session 5 Session 6 Session 7 Session 8	-1.20 -1.69 -1.46 -1.55 -2.09 -1.52 -2.54 -2.60	[-2.36; -0.027] [-2.94; -0.45] [-2.77; -0.14] [-2.82; -0.27] [-3.34; -0.83] [-2.89; -0.15] [-3.88; -1.21] [-3.83; -1.36]	0.045* 0.0078* 0.030* 0.017* 0.0011* 0.029* < 0.001* < 0.001*
Stress			
Time (reference = baseline)			
Session 1 Session 2 Session 3 Session 4 Session 5 Session 6 Session 7 Session 8	-3.33 -4.76 -5.78 -6.79 -8.62 -8.40 -9.12 -8.78	[-5.73; -0.94] [-7.30; -2.22] [-8.44; -3.13] [-9.39; -4.18] [-11.16; -6.08] [-11.18; -5.63] [-11.81; -6.42] [-11.27; -6.29]	0.0064* < 0.001* < 0.001* < 0.001* < 0.001* < 0.001* < 0.001* < 0.001*
Depression			
Time (reference = baseline)			
Session 1 Session 2 Session 3 Session 4 Session 5 Session 6 Session 7 Session 8	-1.40 -1.27 -2.98 -2.89 -3.12 -3.69 -3.93 -4.13	[-2.66; -0.13] [-3.63; -0.90] [-4.40; -1.56] [-4.27; -1.51] [-4.48; -1.76] [-5.17; -2.21] [-5.38; -2.49] [-5.47; -2.81]	0.030* 0.0011* < 0.001* < 0.001* < 0.001* < 0.001* < 0.001* < 0.001*

*p < 0.05

**Fig. 4:**
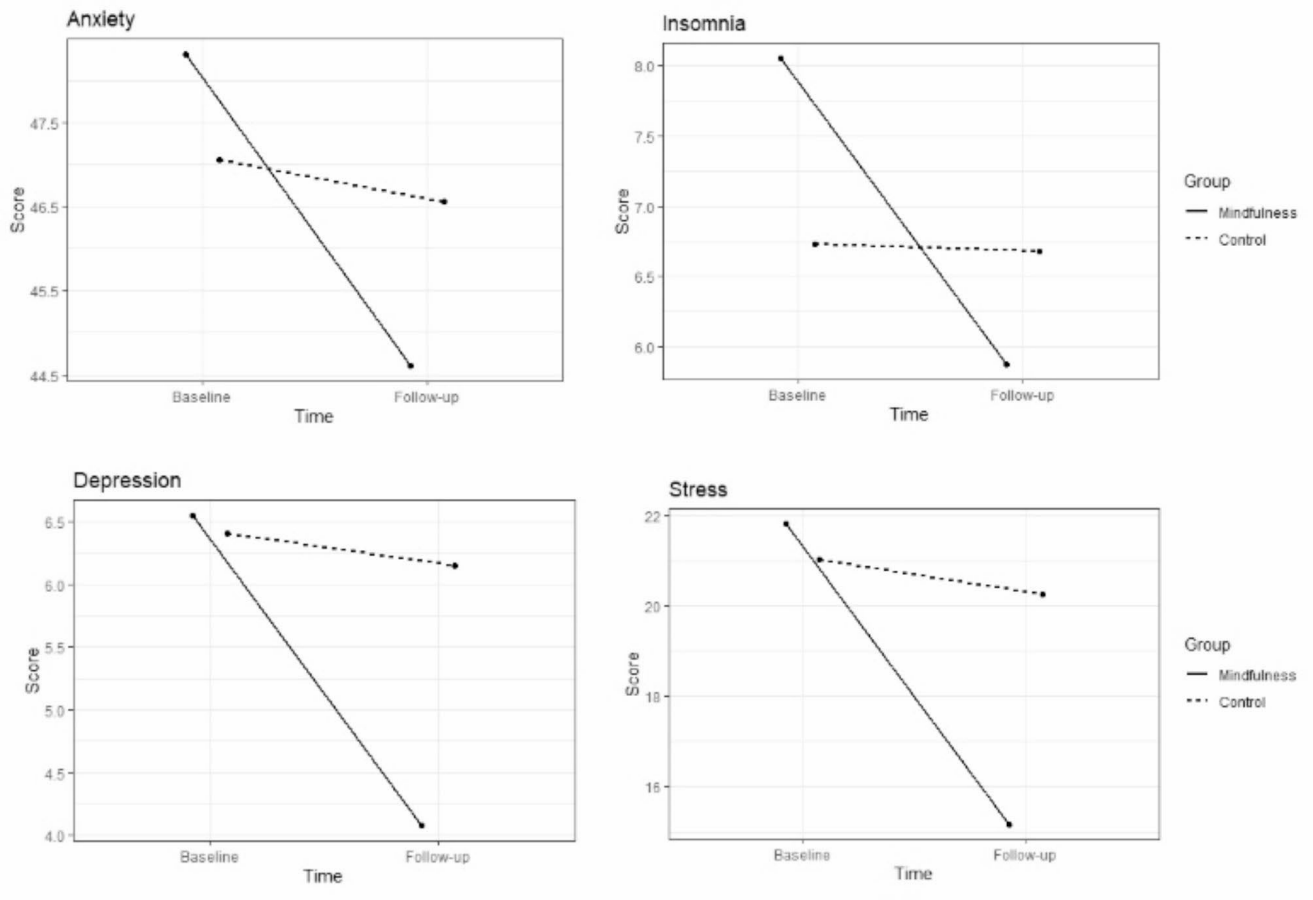
Effect of the MBI on stress (measured by PSS-19), anxiety (measured by STAI-trait), depression (measured by PHQ-9) and insomnia (measured by ISI) over time. Outcome values averaged over the levels of covariates are presented

The effect of the mindfulness-based intervention over time, in respect of mental health issues, was verified for the variables stress (B = 5.88, p < 0.001), depression (B = 1.54, p < 0.001) and insomnia (B = 1.74, p = 0.020). No effect was found for anxiety symptoms (Table 3; Fig. 4).

**Table 3:** Effects of mindfulness-based intervention on self-report instruments (stress, anxiety, depression, insomnia) at baseline and follow-up (n = 136)

	B	[95% CI]	*p*
**PSS (stress)** ^**a**^			
Group (reference = mindfulness)	-0.79	[-3.42; 1.84]	0.560
Time (reference = baseline)	-6.65	[-8.18; -5.11]	< 0.001*
Group*time Control x T1	5.88	[3.12; 4.24]	< 0.001*
**STAI (anxiety)** ^**b**^			
Group (reference = mindfulness)	-1.25	[-3.93; 1.43]	0.360
Time (reference = baseline)	-3.70	[-6.50; -0.91]	0.001*
Group*time Control x T1	3.20	[-0.86; 5.61]	0.120
**PHQ (depression)** ^**c**^			
Group (reference = mindfulness)	0.98	[0.78; 1.23]	0.850
Time (reference = baseline)	0.62	[0.53; 0.74]	< 0.001*
Group*time Control x T1	1.54	[1.20; 1.86]	< 0.001*
**ISI (insomnia)** ^**c**^			
Group (reference = mindfulness)	0.83	[0.66; 1.07]	0.120
Time (reference = baseline)	0.73	[0.62; 0.86]	< 0.001*
Group*time Control x T1	1.74	[1.06; 1.74]	0.020*

* p < 0.05; All models were adjusted by subject’s id (random effect), sex, age and whether or not they were in a exams period. (a) linear model; (b) model with Poisson distribution; (c) model with negative binomial distribution and exponential coefficients.

Contrast tests between pairs showed that there were no differences between groups at baseline in any of the parameters. For stress, significant differences occurred between the mindfulness group at baseline (M = 23.98, SD = 7.75) and T1 (M = 17.27, SD = 7.66) (B = 6.58, SE = 0.79, p < 0.001); control at baseline (M = 23.77, SD = 6.96) and mindfulness at follow up (B = 5.79, SE = 1.39, p < 0.001); and control (M = 22.07, SD = 8.18) and mindfulness at T1 (B = -4.96, SE = 1.48, p = 0.0056). This means that those in the mindfulness group presented a significant difference at T1 both in respect of initial scores and of the second scores from control group.

For depression, the results were similar; differences were found in the pairs: mindfulness at baseline (M = 9.84, SD = 6.24) and T1 (M = 6.20, SD = 5.14) (B = 1.60, SE = 0.13, p = < 0.01); control at baseline (M = 9.42, SD = 5.05) and mindfulness at follow up (B = 1.58, SE = 0.20, p = 0.0029); control (M = 8.69, SD = 5.06) and mindfulness at T1 (B = 0.65, SE = 0.091, p = 0.013). Regarding insomnia, the mindfulness group showed a significant reduction from baseline (M = 10.70, SD = 5.89) to T1 (M = 8.45, SD = 7.37) (B = 1.38, SE = 0.12, p = 0.0018).

## Discussion

Reduced symptoms of depression, stress and insomnia were observed in university students after the application of an adapted version of the MBRP, when compared to the control group. However, it did not modify symptoms of anxiety, differing from what was expected. These results add to the literature in respect of the impacts that this type of intervention can have on the mental health of university students.

Also, during protocol, there were statistically significant differences in the symptoms of stress, depression and insomnia ever since the first section. Although it has not happened consistently and progressively, in general, there was a reduction in these symptoms presented by university students throughout the entire protocol. It is important to highlight that these trends may not be the result of each session directly and all measures were compared to the baseline, but it can be important to explore ways in which the sessions could have influenced the observed results.

A continuous decrease in these symptoms in the first three sessions could be seen. In general, these sessions were about the presentation of the concept and relationship of mindfulness to what is experienced in everyday life. A slight increase in symptoms of depression and insomnia was seen after the fourth session, when challenging situations are discussed and how mindfulness can be used to deal with them. In addition to the effect of the session itself, situations outside the context of the practices may also have contributed to the result found, but the changes were slight. The average of both symptoms dropped again in the following week, after the fifth session.

After the sixth session, in which thoughts and our reactions to them are worked on, there was an increase in stress and insomnia symptoms, while depression symptoms remained stable when compared to the previous week. It is possible that, when turning the attention to the thoughts in an attempt to disengage, but still without the consolidated skill, observation brings discomfort and stress, which can affect sleep.

According to the Monitoring and Acceptance Theory (MAT), awareness of experiences, that is, the ability to monitor attention, can explain the improvement presented with regard to the cognitive functioning of individuals who undergo mindfulness protocols, but this same skill can increase affective reactivity [43], which could explain this slight increase. In addition, the theory postulates that acceptance is essential to modify the individual’s relationship with the monitored experience, reducing affective reactivity and, therefore, leading to the improvement of negative affectivity and symptoms related to stress [43]. Thus, in line with previous studies [44], the present study indicates the possibility that a greater emphasis on acceptance skills, an important emotion regulation mechanism, could maximize the effectiveness of these interventions.

Finally, after the seventh session, when the focus is on self-care, all symptoms showed a visible decrease, which remained until the end of the protocol. One previous study that assessed the weekly change in stress symptoms in patients at a medical centre was found, where a statistically significant difference was observed in relation to the baseline only after the fourth session [45]. Although the weekly assessment has not yet been much explored, modifications in the length of the protocols have already been tested.

A previous study evaluated the effect of a brief mindfulness intervention performed in just three sessions in an adult audience without major psychological symptoms and observed a decrease in the Depression, Anxiety and Stress Scale (DASS) score and an improvement in life satisfaction [46]. Four-session mindfulness interventions have also been previously tested, although there was no consistency between them. While some did not find differences in psychological symptom rating scales after the intervention [47] or compared to the active control group [48], others observed changes similar to the 8-week protocol [49], decreased proinflammatory cytokines related to depression disorder [47], improvements in depression symptoms and of psychological well-being [50] and the reduction of tiredness, anxiety, improvement of visual-spatial processing, working memory and executive functioning [51].

Practicing mindfulness between sessions is already known as an important factor for the number of positive consequences observed [52, 53]. However, many students informally claim, throughout the sessions, not finding the time or even remembering to do the practices, especially when the university demands more from them (examination periods, assignments, etc.). The use of cell phone applications to send daily reminders for the personal practice of individuals has already been proposed, having been presented as a viable possibility and well accepted by university students [54, 55]. The use of technology combined with the weekly protocol can be a way to further expand the gains obtained by the practice of mindfulness, and future studies are needed to assess how effective the use of this type of tool within the protocol is and whether the weekly gains are also necessarily related to that individual practice.

Thus, the need for further studies to explore the moment when changes happen is highlighted, in order to better understand what the possible modifications are in the protocols. This information could help both cultural and specific adaptations for different populations, as well as changes in the number of sessions of interventions.

Corroborating the initial hypotheses, symptoms of depression presented by the students decreased significantly after the 8-week MBI protocol. This result is in line with the literature, where improvements in these symptoms have already been reported through MBIs in several populations [56, 57] and also in meta-analyses [58, 59]. However, mindfulness practices might act upon different cultures in different ways due to their different characteristics and baseline scores [60]. Therefore, it is fundamental to keep studying its effects on unexplored or under-explored populations, such as Brazilian university students.

Although none of the participants declared a sleep disorder, the insomnia severity questionnaire pointed to a low quality and quantity of sleep at baseline. This may not have been perceived by students as a problem or been identified as a sleep disorder. Following the intervention, there was an improvement in insomnia symptoms, which might indicate the impact that mindfulness can have on the lives of its practitioners. A lower quality and quantity of sleep have already been shown to be associated with lower academic performance and quality of life, decreased cognitive functions and an increased propensity to develop mental disorders [60–64]. Also, it may have consequences that have been singly addressed, such as the symptoms of depression and the increased level of stress. Thus, it is possible that the improved sleep of individuals who underwent the protocol can impact other aspects of their lives. Future studies are needed to investigate this hypothesis.

The protocol used did not specifically focus on stress, but students also reported a decrease in perceived stress after the intervention. Stress is a significant health challenge in the 21st century, with many people developing disorders and having a reduced quality of life due to this instinctive response proving to be maladaptive in some modern contexts. The effect of MBIs on stress has been reported for decades in different populations [64–67]. A recent study indicated that stress reduction mediated the positive relationship between mindfulness and well-being in the general population [68], pointing to a central role of this construct in the analysis of the effects of the practice.

However, contrary to our expectations, the students’ anxiety symptoms did not improve after the mindfulness protocol when compared to the control group. In fact, there is no consensus in the literature on the effect of mindfulness on anxiety, with some studies pointing to improvements [67, 69] and others, including ours, finding no difference [65, 70]. A meta-analysis of the effect of MBIs on anxiety among college students reported a significant improvement, although there is a chance that the result was influenced by publication bias [71]. Precisely due to this bias, the importance of reporting the results found in the present study is highlighted. The lack of a significant difference may be the result of several other factors, such as scale sensitivity, since the aforementioned meta-analysis excluded studies that addressed anxiety as a trait because of its greater stability while we chose to use it exactly for this reason; differences in protocols; and the socio-political context of the research, as it was carried out at public universities in a troubled political period with scholarships and grants being cancelled, which may have affected the participants’ feelings of insecurity and anxiety.

One important observation made when conducting the research was that students were very interested in taking part in mindfulness practices. Despite this initial interest, there were a high number of dropouts from the groups. Future researches are necessary in order to better explore the reasons why students present such a high rate of dropout.

The study has limitations that should be considered. The use of self-administered questionnaires limits objectivity, making it necessary to use multiple methods to overcome this issue. The high number of dropouts during the protocol, despite being common and within expectations, affects the results of the study and needs to be taken into account. A longer follow-up time after the intervention would be beneficial in investigating more long-term effects. The research sample contained a large disproportion between genders, with 80% being female, and therefore limiting the generalization of the results to men, although previous studies have suggested that the relationship between mindfulness and mental health is similar between genders [72, 73]. Moreover, the wait-list control group was not active; as meeting in groups and taking time for yourself (regardless of the use of particular interventions) may impact mental health, it is imperative to evaluate these outcomes in clinical trials using active control groups.

Despite these limitations, the present study makes an important contribution to the literature on interventions to improve the mental health of university students, adding longitudinal data from a population that is still little explored in Brazil. In addition, results from an increasing diversity of populations, settings and cultures can enhance the understanding of the benefits and limits of mindfulness practices. This study also makes it possible to analyse the symptoms throughout the intervention, enabling a better understanding of when and how the changes occur. Finally, this study makes an important contribution to the understanding of how a relatively brief approach (8-weeks protocol) can impact a population that contains a high number of individuals who may suffer with mental health issues.

It is important to emphasize that further studies with a longer T1 time are required to observe the protocol’s long-term effects. Moreover, it could be of interest to evaluate whether MBIs have an impact on seeking psychological support, and the viability of implementing MBIs’ protocols in universities as a prevention program in order to prevent the development of mental disorders.

## Conclusion

It was observed a decrease in the symptoms of mental health disorders in university students after its participation in a MBI intervention focusing on reactivity and health promotion, an adaptation of MBRP protocol. Addressing mental health problems before the development of disorders, and even in the presence of it, in university students is an important step towards a healthier and more productive higher educated population. Mindfulness practices could be further investigated and used as a potential investment in this area, enabling health education, health promotion and mental disorders prevention among university students, regardless of the chosen protocol, as the improvements related to symptoms of mental health problems seem to be transversal to all.

## Data Availability

The datasets analysed during the current study are available from the corresponding author on reasonable request.
